# Esomeprazole inhibits hypoxia/endothelial dysfunction–induced autophagy in preeclampsia

**DOI:** 10.1007/s00441-022-03587-z

**Published:** 2022-01-29

**Authors:** Shengyi Gu, Chenchen Zhou, Jindan Pei, Yuelin Wu, Sheng Wan, Xiaobo Zhao, Junhao Hu, Xiaolin Hua

**Affiliations:** 1 Shanghai First Maternity and Infant Hospital, School of Medicine, Tongji University, Shanghai, 200092 China; 2 Interdisciplinary Research Center On Biology and Chemistry, Shanghai Institute of Organic Chemistry, Chinese Academy of Sciences, Shanghai, 201203 China

**Keywords:** Placenta, Preeclampsia, Autophagy, Esomeprazole, AMPKα-mTOR

## Abstract

Preeclampsia (PE) affects 3 to 5% of pregnant women worldwide and is associated with fetal and maternal morbidity and mortality. Although a complete understanding of PE remains elusive, it has been widely accepted that a dysfunction of the placenta plays a key role in the pathogenesis of PE. In this study, we investigated the role of excessive placental autophagy during PE pathogenesis and explored whether esomeprazole ameliorates PE by inhibiting the autophagy in the placenta. The PE cellular model was established by treating the cells’ L-NAME and hypoxia. The PE mice model was established by L-NAME administration and was confirmed by the increased systolic blood pressure (SBP) and urinary protein detected. The autophagy and key proteins were detected in human placental tissue, in cells, and in the mice model by Western blot and immunofluorescence staining. Results showed that excessive autophagy could be detected in human PE placental tissue, in the PE cellular model, and in the PE mice model. Hypoxia induces autophagy by activating AMPKα and inhibiting mTOR in vivo and in vitro. Esomeprazole inhibits L‐NAME-induced autophagy in mice by inhibiting AMPKα and activating mTOR. In conclusion, this study demonstrates that the excessive autophagy induced by the SIRT1/AMPKα-mTOR pathway plays a significant role in the pathogenesis of PE. However, esomeprazole treatment inhibits AMPKα but activates mTOR, resulting in the inhibition of autophagy in the placenta and, therefore, mitigates PE symptoms.

## Introduction

Preeclampsia (PE), characterized by hypertension and proteinuria in mid- or late-term pregnancy, is one of the most serious pregnancy complications, causing multiorgan injury (Mol et al. 2016a). Preeclampsia affects 3–5% of pregnancies worldwide and has been closely associated with fetal and maternal morbidity and mortality (Rana et al. 2019; Mol et al. 2016a).

An initial asymptomatic phase during the first trimester of gestation is widely accepted as the pathogenesis of preeclampsia, which is characterized by a deficient trophoblast invasion and spiral artery recasting disorder (Phipps et al. 2019), followed by anoxia and malnutrition of the placenta, resulting in hypertension and proteinuria. Moreover, the maternal–fetal interface shows inflammatory overactivation and endothelial dysfunction. The placenta plays a vital role in the pathogenesis of preeclampsia.

Hypoxia, undernutrition, inflammatory overactivation, and endothelial dysfunction can induce autophagy (Sasaki et al. 2007; Hiby et al. 2010). Autophagy is an intracellular self-degrading system characterized by a widespread degradation process for long-lived proteins or cytoplasmic components during undernutrition (Nakashima et al. 2017; Glick et al. 2010). Autophagy’s regulatory mechanism is complex, and its upstream signaling pathway mainly involves an mTOR-dependent pathway and an mTOR-independent pathway (AMPK, PI3K, Ras-MAPK, p53, PTEN, endoplasmic reticulum stress). Cells use macroautophagy/autophagy to facilitate survival by maintaining cellular integrity when experiencing strong environmental stimuli such as hypoxia (Mazure and Pouysségur 2010). In the case of hypoxia, HIF-1α and HIF-2α are the mediators of the hypoxic stress signal. The HIF-dependent induction of autophagy by hypoxia has been reported. Upon a hypoxia-mediated autophagy induction, the HIF-1α-eliminated cells showed a decrease in the levels of the well-known autophagy markers beclin-1 and LC3B-II (Ravanan et al. 2017). HIF activates autophagy through BNIP3 (Bcl-2/E1B 19 kDa-interacting protein 3). The HIFα and IKK mediate the inflammatory and hypoxic signals, activating a cascade of signaling them downstream to activate inflammation and autophagy as stress responses (Bellot et al. 2009). The NF-κB links autophagy and inflammation via HIFα. The mTOR inhibition activates the NF-κB in the inflammatory pathway, while the mTOR activation initiates the transcriptional activity of HIFα. Additionally, autophagy is necessary for embryonic development and placenta implantation in mammals (Boya et al. 2018; Nakashima et al. 2019). For placental villi bathed in maternal blood, once the placenta is in an autophagy disorder, abundant toxic factors, such as soluble Fms-like tyrosine kinase-1(sFlt-1), are released and damage maternal vasculature (James et al. 2005). Thus, researchers have devoted increasing attention to the role of autophagy in the placenta.

The termination of the pregnancy is the first choice for the treatment of preeclampsia, which potentially generates iatrogenic prematurity in babies and affects neonatal outcomes (Tomimatsu et al. 2019). To prolong pregnancy, there is no treatment for the pathogenesis except for symptomatic treatment. Esomeprazole, a proton-pump inhibitor, is used to treat reflux esophagitis and hyperemesis gravidarum (Malfertheiner et al. 2017). It has been found to significantly reduce the levels of sFlt-1 and sENG and to effectively prolong pregnancy duration (Onda et al. 2017). Esomeprazole also mitigates endothelial dysfunction by inhibiting the expression of tumor necrosis factor α, vascular cell adhesion molecule 1, and endothelin 1 (Sandrim et al. 2019). A randomized placebo-controlled trial found that esomeprazole could prolong gestation three more days compared to the placebo in pregnancies with preterm preeclampsia (Cluver et al. 2018). Therefore, it has been suggested as a potential drug for treating preeclampsia. However, the mechanisms of esomeprazole in placental function remain unclear.

In this study, we report that normal autophagy was necessary for placental function. We investigate the effect of excessive autophagy and the effect of esomeprazole on PE. Finally, we find that esomeprazole inhibits hypoxia/endothelial dysfunction–induced autophagy via the AMPK/mTOR pathway.

## Materials and methods

### Placental tissue collection

Human placental tissue was collected under appropriate Human Research and Ethics Committee approvals (KS1957). Written and informed consents were obtained from each patient before surgery.

One cubic centimeter of placental tissue was removed from the maternal side of the placenta (3 cm from the edge in the 3, 6, 9, and 12 o’clock directions). The PE group was selected based on relevant recommendations by the Chinese Medical Association. The control placental tissues were obtained from age-matched preterm pregnancies with normally developing fetuses that did not have signs of hypertension disorder or other pregnancy-related diseases.

### Cell line cultures

Human chorionic trophoblast cells (HTR-8/Svneo) seeded at a density of 2 * 10^6^ cells into 10-cm dishes were kept at 37℃ in 5% CO_2_ and 20% O_2_ and cultured in 1640 media (Gibco) with a 10% Australian fetal bovine serum.

Human umbilical vein endothelial cells (HUVECs) seeded at a density of 2 × 10^6^ cells into 10-cm dishes were kept at 37 ℃ in 5% CO_2_ and 20% O_2_ and cultured in ECM media (Sciencell) with a 5% Australian fetal bovine serum and EGF.

When performing hypoxia, L-NAME (MCE, HY-18729), and esomeprazole (Sigma-Aldrich, PHR-1585) treatments, HTR-8/Svneo and HUVEC cells were treated with and without either L-NAME (70 mM) or esomeprazole (50 mM) and hypoxia for 48 h.

All cell experiments were repeated three times.

### PE mice model

Eight‐week-old female C57BL/6 mice (weighing 25–30 g) were used in this study. Male and female mice were mated in the same cage at a male-to-female ratio of 1:2. The time at which the vaginal plug was found was defined as GD0.5. Subsequently, pregnant mice were divided into three groups: the control group (normal pregnancy, *n* = 5), PE group (*n* = 5), and treated-PE group (esomeprazole 40 mg/kg, *n* = 5). The PE and treated-PE groups were treated by continuous treatment with L‐NAME dissolved in drinking water (1 g/L 10 mL/day; Sigma) beginning from GD7.5 to GD18.5. From GD7.5 to GD18.5, esomeprazole dissolved in 100 µl 1% DMSO (*v*/*v*) was injected intraperitoneally for the treated-PE group every day, and an equal volume of 1% DMSO (*v*/*v*) was injected intraperitoneally for the PE and control groups each day. At GD20, the mice were sacrificed, and the placenta and blood specimens were collected. All experimental protocols were approved by the ethics committee at Xinhua Hospital, Shanghai Jiaotong University School of Medicine.

### Assessment of systolic blood pressure

The systolic blood pressure (SBP) of each mouse was measured noninvasively at GD18.5 via the Mouse and Rat Tail Cuff Blood Tail‐Cuff Device (American IITC). Before each measurement was taken, each mouse was exposed to 37 ℃ for 5 min; measurements were taken three times for averaging purposes.

### Placental histology

Placenta specimens from the separate groups were preserved in tissue fixatives, embedded in paraffin after 24 h, and cut into 5-µm sections. After hematoxylin and eosin staining and Masson trichrome staining, morphological evaluations were performed under light microscopy.

### Western blot

Human tissue from control and PE placenta and cell lysates from HTR-8/Svneo cells, HUVEC cells, and mouse placentas from the control, PE, and treated-PE groups were detected by Western blotting, as previously described (Gu et al. 2019). Proteins extracted from tissue and cell samples were fractionated on sodium dodecyl sulfate‐polyacrylamide gel and electrophoresis gels and transferred to polyvinylidene fluoride membranes. Primary antibodies against β-actin (8427 s, rabbit [WB 1:1000]), phospho-AMPKα (2535, rabbit [WB 1:1000] [IF 1:100]), AMPKα (5831, rabbit [WB 1:1000]), PPARγ (2435, rabbit [WB 1:1000]), SIRT1 (2496, rabbit [WB 1:1000]), mTOR (2983, rabbit [WB 1:1000]), phospho-mTOR (5536, rabbit [WB 1:1000]), p53 (2527, rabbit [WB 1:1000] [IF 1:100]), SQSTM1/p62 (7695, rabbit [WB 1:1000] [IF 1:100]), LC3B (3868, rabbit [WB 1:1000] [IF 1:100]), and PI3 kinase class III (4263, rabbit [WB 1:1000]) were purchased from CST. The primary antibodies against HIF-1α (ab1, mouse [WB 1:1000]) and secondary antibodies were HRP-linked using goat anti-rabbit and goat anti-mouse IgG antibodies (CST, 7074, 7076). The bands were visualized using an ECL kit. Band densitometries in equiconditional area were evaluated by ImageJ. The intensity of both the target protein bands and corresponding internal reference bands was quantified. The intensity value of target protein bands was normalized to the value of the reference bands.

### Immunofluorescence staining

Immunofluorescence staining was performed as previously described. Placenta specimens in distinct groups were preserved in tissue fixatives, embedded in paraffin after 24 h, cut into 5-µm sections, and permeabilized with 0.2% Triton X-100 in PBS. HTR-8/Svneo cells and HUVEC cells were fixed with 4% PFA for 15 min and permeabilized with 0.2% Triton X-100 in PBS. Placental sections and cells were precultured in a blocking solution at room temperature to block nonspecific binding sites. They were then cultured with primary antibodies overnight at 4 ℃. Placental sections and cells were treated with DAPI (40, 6-diamino-2-phenylindole) for nuclear detection. Fluorescence images were observed and captured using confocal microscopy.

### Statistics

The data are represented as mean ± SD. One-way analysis of variance (ANOVA) was employed for multiple comparisons, followed by Turkey post hoc testing using SPSS 23.0 and GraphPad Prism 9. A *P* value or adjusted *P* value less than 0.05 was considered to be statistically significant.

## Results and discussion

### Results

#### Increased autophagy in placentas of PE patients

**Fig. 1:**
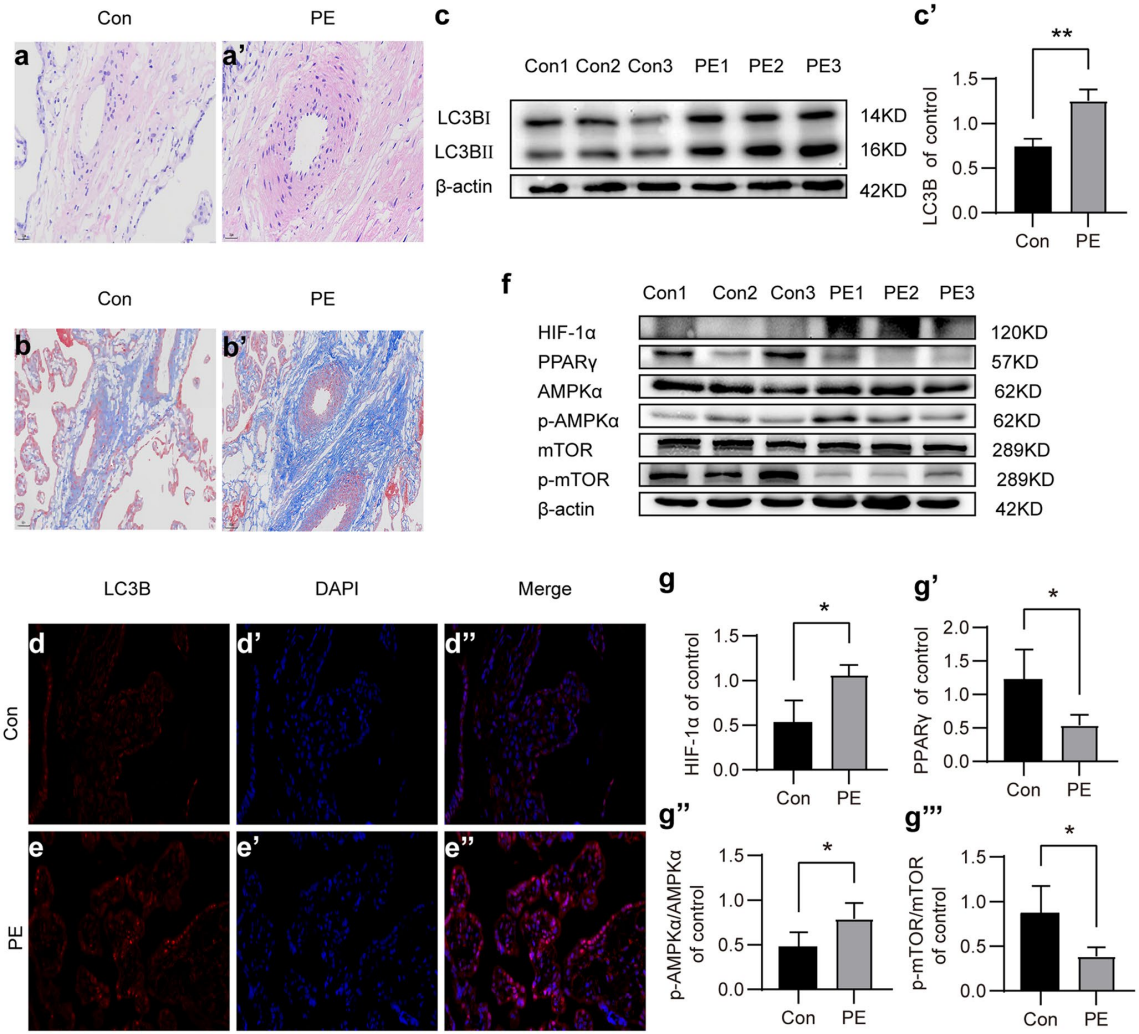
Increased autophagy in placentas of preeclampsia. (**a**, **a′**) Histology analysis of H&E-stained healthy and PE placentas. (**b**, **b′**) Trichrome Masson staining showing increased fibrosis in PE placenta. (**c**) The expression levels of LC3B and beta-actin were analyzed using Western blot. (**c′**) Plot showing the quantification of relative LC3B expression levels in healthy and PE placentas. Beta-actin expression levels were used for normalization. (**d**–**e″**) The expressions of LC3B in placentas were validated using immunofluorescence staining. (**f**) The expression levels of the essential autophagy-related proteins HIF1α, PPARγ, AMPKα, p-AMPKα, mTOR, and p-mTOR were measured using the Western blot test. (**g**–**g‴**) Plots showing the quantification of HIF1α, PPARγ, p-AMPKα/AMPKα, and p-mTOR/ mTOR. All data are representative of three independent experiments. *P < 0.05, **P < 0.01

We first histologically analyzed the structure of healthy and PE placentas. H&E and Trichrome Masson’s staining of placenta sections showed that PE placentas showed abnormal structure compared to healthy controls, including reduced branching, compaction of the labyrinth area, increased fibrosis, and reduced vascularization (Fig. 1a–b′). Previous studies have suggested that autophagy may play a role in PE pathogenesis; therefore, we analyzed the LC3B, a well-characterized marker of autophagy, and protein levels in placentas using the Western blot. Results showed that the LC3B expression was significantly increased in the PE placentas than in the control group (Fig. 1c, c′, *P* < 0.05). The immunofluorescence-staining analysis of PE and healthy placentas further confirmed the elevated expression of LC3B in PE placentas (Fig. 1d–e″, *P* < 0.05).

#### Hypoxia induces autophagy by activating AMPKα and inhibiting mTOR in PE human placenta

Interestingly, Western blot analysis showed that the HIF-1α protein level was significantly increased in PE placenta compared to that in the control group (Fig. 1f, g′, *P* < 0.05), indicating that PE placentas were in a state of hypoxia. Further analysis showed that the phosphorylation level of AMPKα was significantly elevated (*P* < 0.05). On the contrary, the phosphorylation of mTOR was significantly reduced (*P* < 0.05) (Fig. 1f–g‴). These data suggest that hypoxia induces autophagy by activating AMPKα and inhibiting mTOR in PE human placenta.

#### Esomeprazole inhibits hypoxia-induced autophagy in vitro

**Fig. 2:**
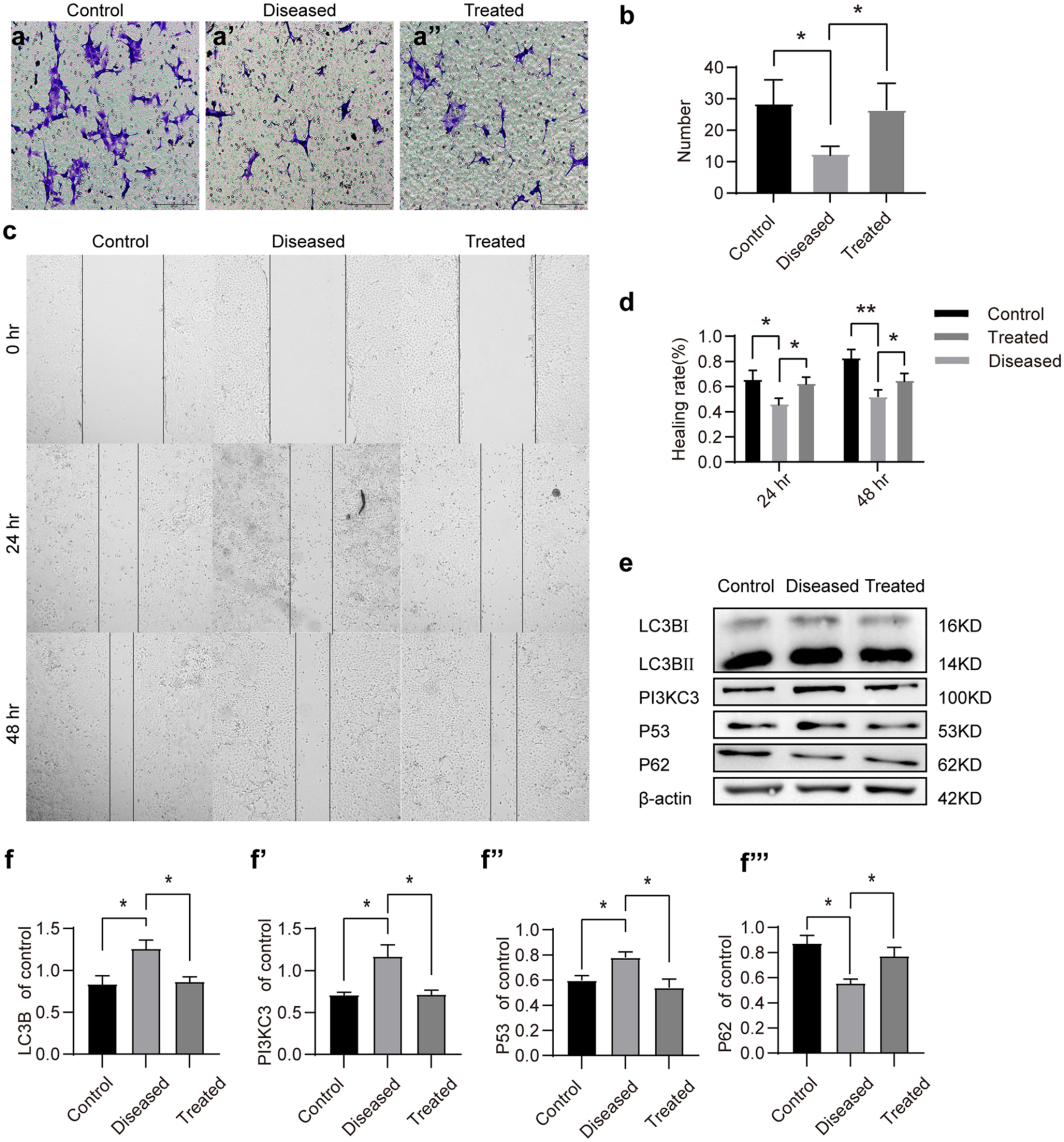
Esomeprazole suppresses migration and autophagy in HTR-8/Svneo cells treated with hypoxia and L-NAME. (**a**, **a′**, **a″**) HTR-8/Svneo cells cultured in transwells were first treated with hypoxia and L-NAME, followed by DMSO or esomeprazole treatment. Transmigrated cells were stained with crystal violet and imaged. (**b**) Plot showing the quantification of the transmigrated cells. (**c**) HTR-8/Svneo cells cultured in 6-well plates. After introducing a cell-free gap, the cells were treated with hypoxia and L-NAME, followed by DMSO or esomeprazole treatment. Cell migrations were recorded at 24 h and 48 h. (**d**) Plot showing the quantification of the healing rate. (**e**) LC3B, P53, and PI3KC3 proteins levels were detected via Western blot. (**f**–**f‴**) Plot depicting the quantification of LC3B, P53, and PI3KC3 expression levels. *P < 0.05, **P < 0.01

**Fig. 3:**
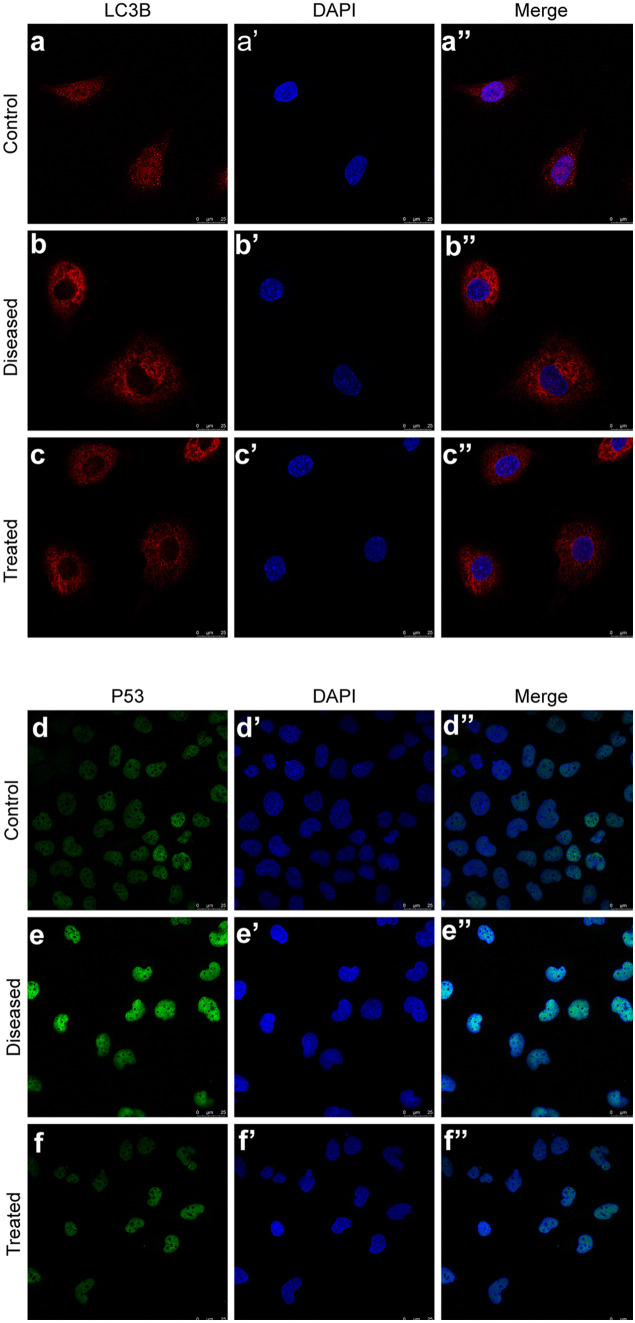
Esomeprazole suppresses autophagy in HTR-8/Svneo cells treated with hypoxia and L-NAME. (**a**–**f″**) The expressions of LC3B and P53 were measured by immunofluorescence staining. All data are representative of three independent experiments

**Fig. 4:**
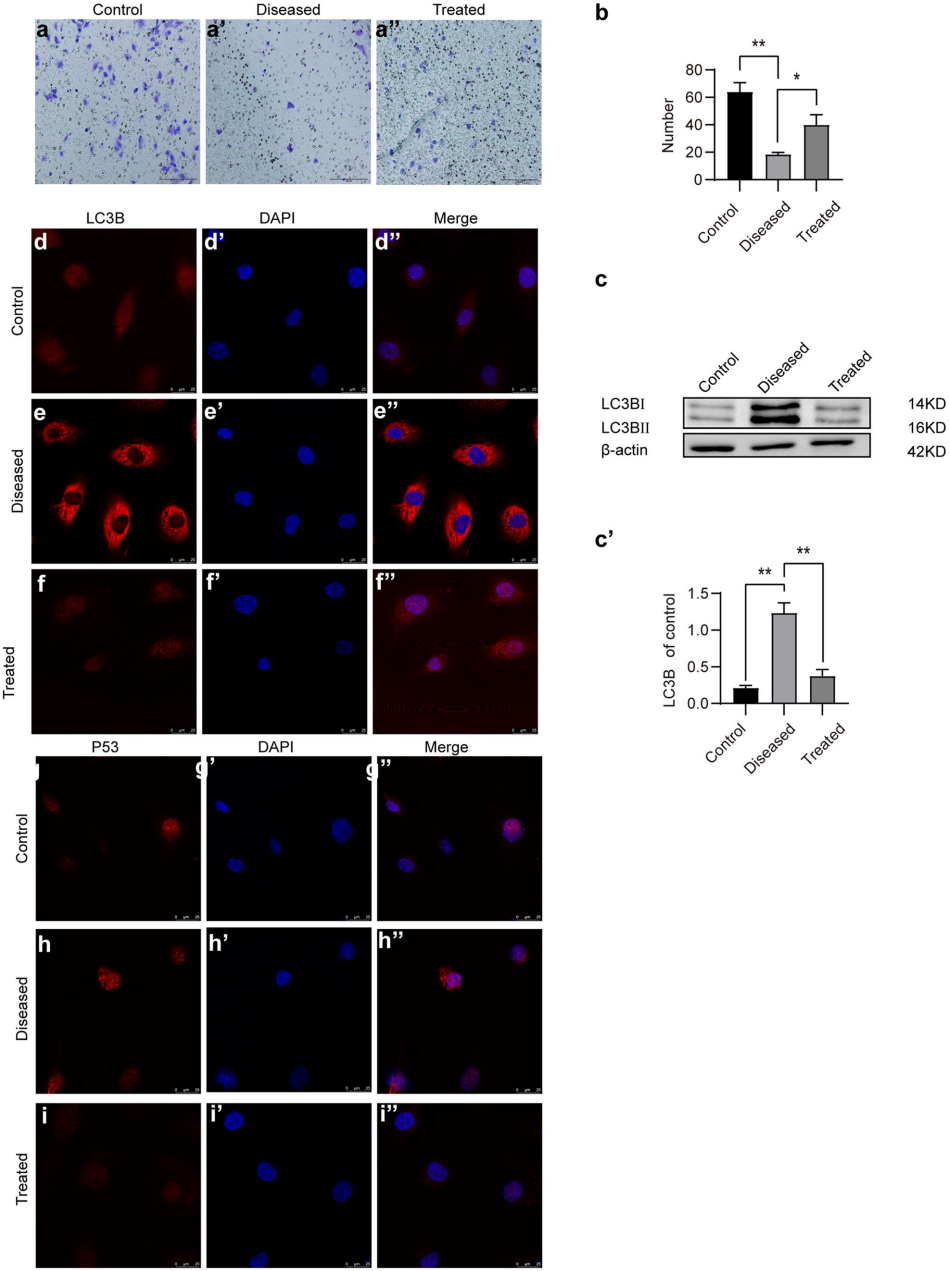
Esomeprazole suppresses autophagy in HUVEC treated with hypoxia and L-NAME. (**a**, **a′**, **a″**) Cell migrations upon hypoxia and L-NAME treatment were measured using transwell-migration assays. (**b**) Quantification of cell migration of (**a**, **a′**, **a″**). (**c**) LC3B proteins levels were detected via Western blot. (**c′**) Plot depicting the quantification of LC3B expression levels. (**d**–**i″**) The LC3B and P53 expressions were visualized using immunofluorescence staining. All data are representative of three independent experiments: *P < 0.05, **P < 0.01

We first tested whether esomeprazole could inhibit autophagy using a cell-based assay approach. Both the transwell migration and wound-healing assay showed that hypoxia and L-NAME (70 µM, 48 h) treatment inhibited the migration of HTR8/SVneo cells (*P* < 0.05). Esomeprazole treatment strongly rescued the cell migration of HTR8/SVneo cells (*P* < 0.05) (Fig. 2a–d). Western blot analysis revealed that LC3B protein levels were elevated in HTR8/SVneo cells subject to hypoxia and L-NAME treatment (70 µM, 48 h), compared to cells under normoxia environment (*P* < 0.05). Concurrently, the expressions of PI3KC3 and P53 also were increased under hypoxia and L-NAME treatments. Nonetheless, esomeprazole treatment successfully reduced LC3B, P53, and PI3KC3 expression in hypoxia/L-NAME-treated HTR8/SVneo cells (Fig. 2e–f‴). This was further confirmed by the immunofluorescence staining analysis of LC3B and P53 (Fig. 3a–f″). In addition to HTR8/SVneo cells, we further validated these findings in HUVECs. As expected, HUVECs treated with hypoxia and L-NAME showed impaired cell migration ability, while the esomeprazole treatment effectively restored HUVEC migration (Fig. 4a, b). Moreover, esomeprazole potently suppressed hypoxia and L-NAME treatment-upregulated LC3B and P53 expression (Fig. 4c–i″). Collectively, these data showed that esomeprazole can abrogate hypoxia and L-NAME-induced autophagy.

#### Esomeprazole reduces autophagy by inhibiting phosphorylation of AMPKα in vitro

**Fig. 5:**
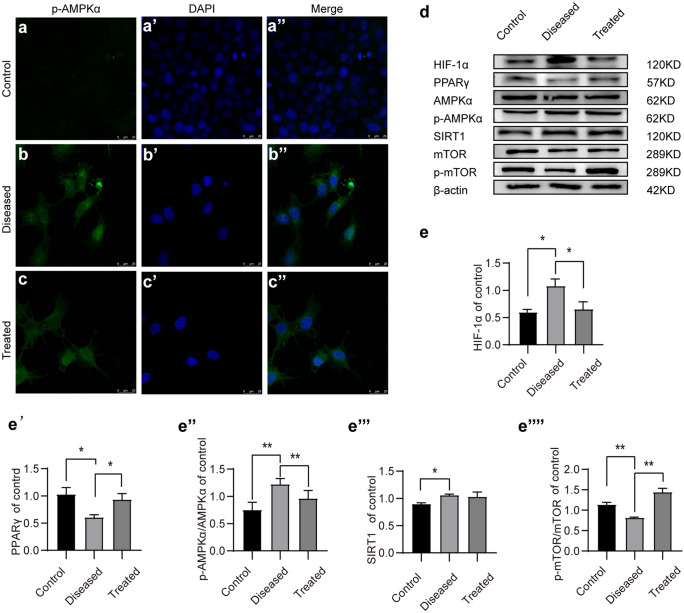
Esomeprazole inhibits autophagy in HTR-8/Svneo cells by suppressing the SIRT1/AMPKα pathway. (**a**–**c″**) Immunofluorescence staining of p-AMPKα in control or esomeprazole-treated HTR-8/Svneo. (**d**) Western blot analysis of the expression levels of p-AMPKα, AMPKα, PPARγ, SIRT1, mTOR, and p-mTOR in control or esomeprazole-treated HTR-8/Svneo. (**e**–**e⁗**) Plots showing the quantification of HIF1α, PPARγ, p-AMPKα/AMPKα, and p-mTOR/ mTOR. All data are representative of three independent experiments: *P < 0.05, **P < 0.01

**Fig. 6:**
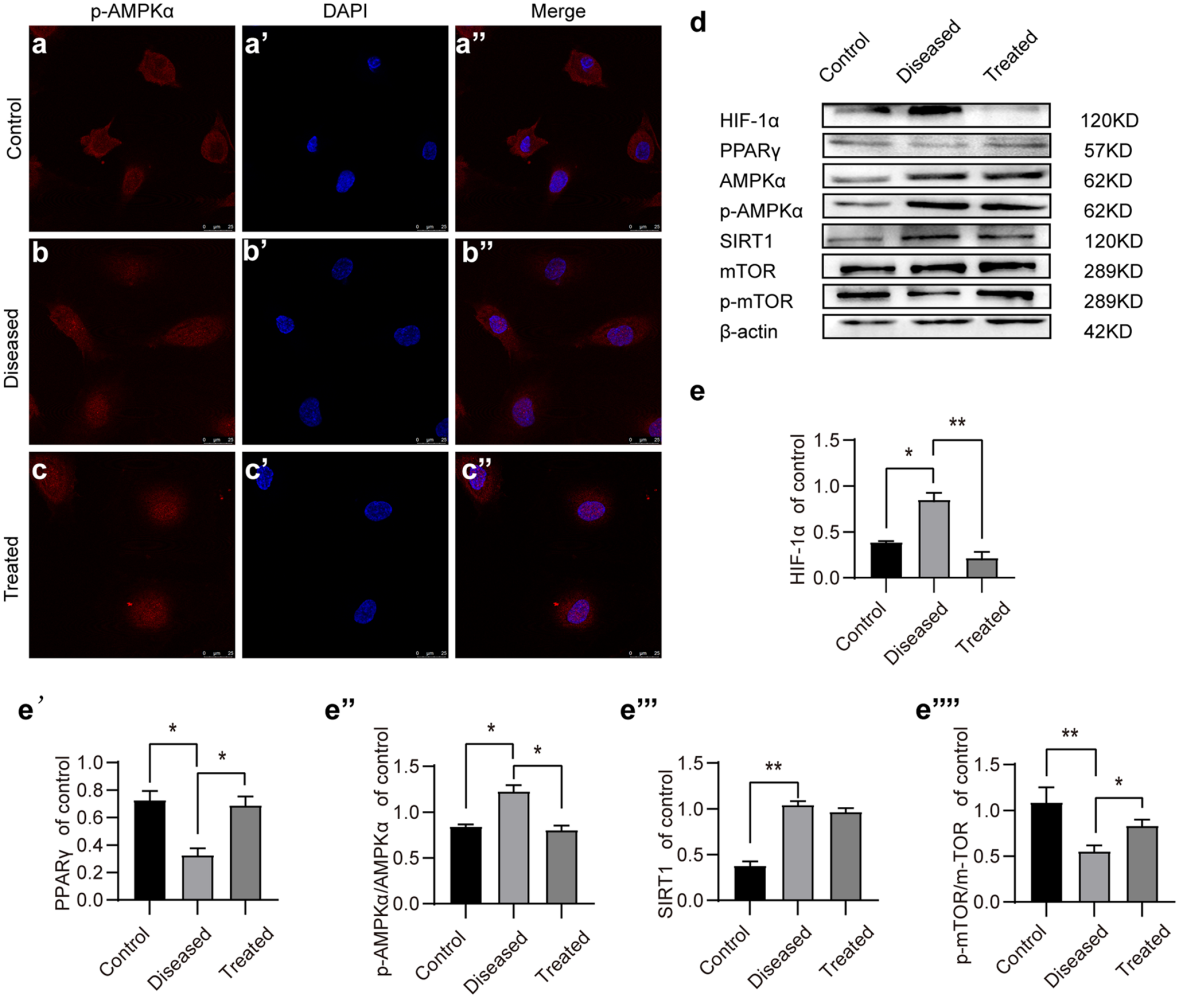
Esomeprazole inhibits autophagy via SIRT1/AMPKα pathway in HUVECs. (**a**–**c″**) Expression levels of p-AMPKα in HUVECs were visulized by immunofluorescence staining. (**d**) The protein levels of HIF1α, phospho-AMPKα, AMPKα, PPARγ, SIRT1, mTOR, and phospho-mTOR were measured using Western blotting. (**e**–**e⁗**) Plot showing the quantification of HIF1α, PPARγ, SIRT1, and phospho-mTOR protein levels in HUVECs. All data are representative of three independent experiments: *P < 0.05, **P < 0.01

Compared to the phosphorylation of AMPKα under normoxia, the phosphorylation of AMPKα was elevated in HTR8/SVneo cells under hypoxia and L-NAME treatment (70 µM, 48 h) (Fig. 5a–d). On the contrary, the phosphorylation of mTOR was significantly inhibited (Fig. 5d–e‴). Notably, esomeprazole treatment reduced AMPKα phosphorylation and SIRT1 expressions, while the phosphorylation of mTOR was significantly increased (Fig. 5d–e‴). We also validated these findings in HUVECs Similar to HTR8/SVneo, HUVECs under hypoxia and L-NAME treatment showed significantly increased phospho-AMPKα and HIF1α levels and decreased PPARγ and phospho-mTOR levels, while they were restored by esomeprazole treatment (Fig. 6a–e‴). Therefore, these results suggest esomeprazole-inhibited hypoxia and L-NAME treatment-induced autophagy by activating mTOR and inhibiting AMPKα signaling.

#### Esomeprazole ameliorates preeclampsia-like symptoms in mice induced by L‐NAME treatment

**Fig. 7:**
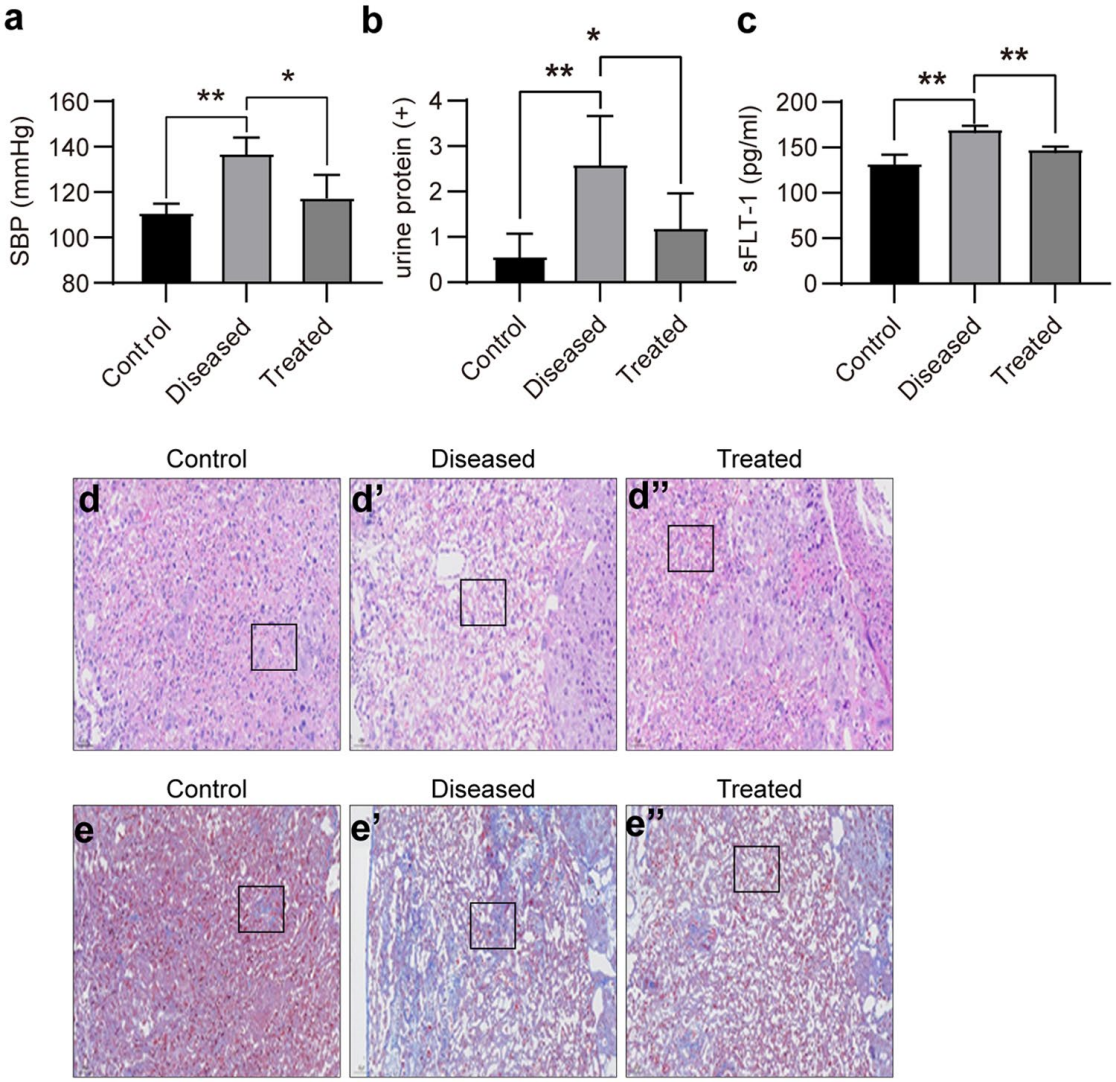
Esomeprazole ameliorates the preeclampsia symptoms caused by L‐NAME. (**a**) Plot showing the SBP of control, L-NAME-treated, or L-NAME + esomeprazole-treated mice were measured. (**b**) Plot showing the urinary protein concentrations of the control, L-NAME-treated, or L-NAME + esomeprazole-treated mice were determined. (**c**) The sFLT-1 concentrations in the serum of control, L-NAME, or L-NAME + esomeprazole-treated mice were determined at GD18.5 using ELISA. (**d**, **d′**, **d″**) Histological analysis of the placentas of control, L-NAME-treated, or L-NAME + esomeprazole-treated mice using H&E staining. (**e**, **e′**, **e″**) Fibrosis in the placentas of control, L-NAME-treated, or L-NAME + esomeprazole-treated mice were measured using Trichrome Masson staining. A blue color in the square indicates collagen deposition, *N* = 3 mice per group, *P < 0.05, **P < 0.01

PE is characterized by significantly increased systolic blood pressure and the onset of proteinuria. The administration of L-NAME successfully led to increased systolic pressure and the presence of protein in the urine (Fig. 7a, b). In addition, L-NAME treatment also increased the sFLT-1 level in serum (Fig. 7c). Esomeprazole treatment significantly reduced systolic pressure, urinary protein, and serum sFLT-1 levels. Furthermore, both H&E-based histology analysis and Trichrome Masson’s staining demonstrated that the esomeprazole treatment effectively preserved placenta structure and reduced intraplacental fibrosis (Fig. 7d–e″). These data strongly indicate that PE symptoms were reversed by esomeprazole treatment.

#### Esomeprazole protects the placenta from L‐NAME-induced autophagy by upregulating PPARγ in vivo

**Fig. 8:**
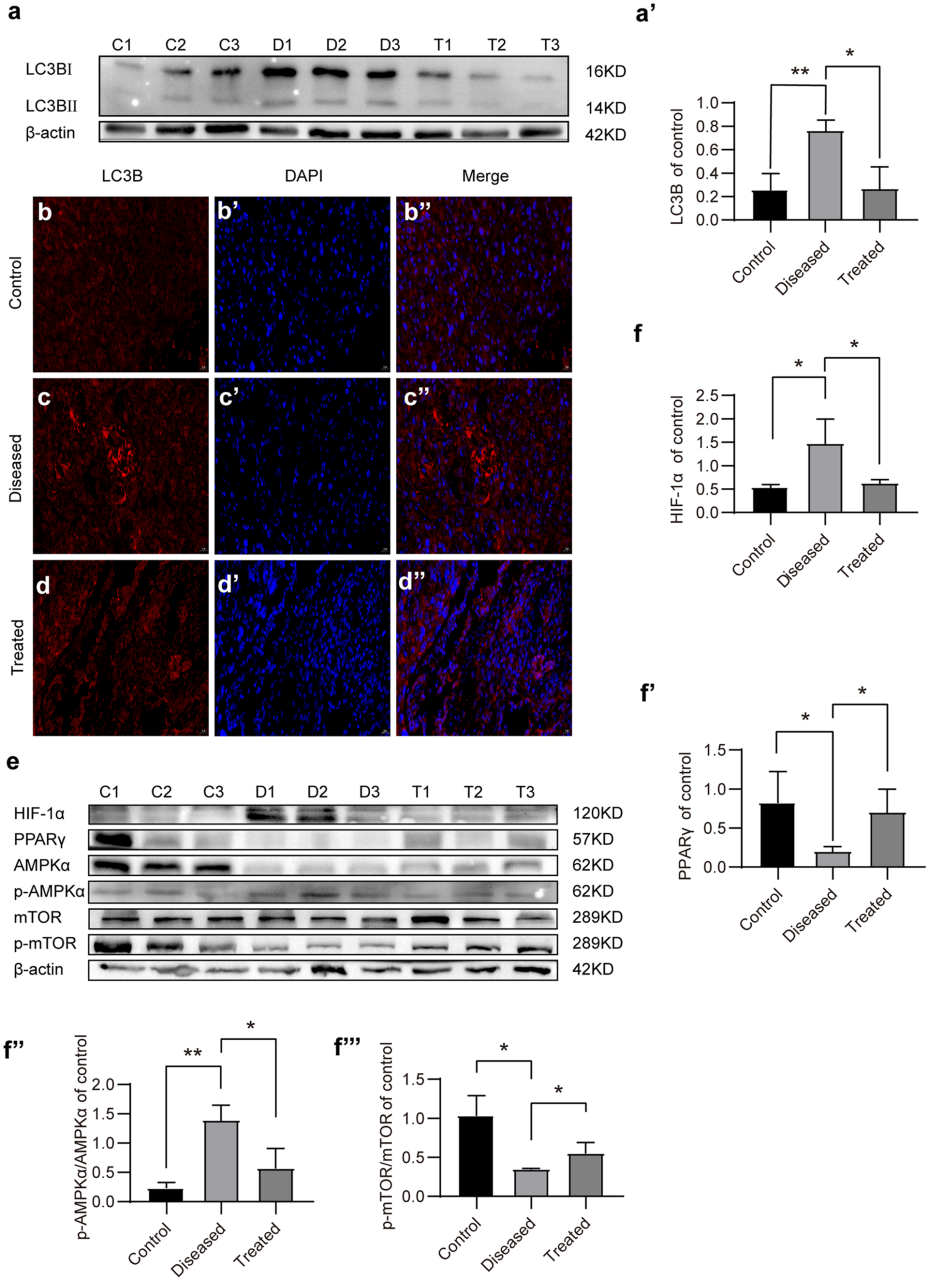
Esomeprazole-suppressed autophagy induced by L-NAME via AMPKα-mTOR in PE mice. (**a**) LC3B proteins levels in placentas of control, L-NAME-treated, or L-NAME + esomeprazole-treated mice were detected by Western blot. (**a′**) Plot depicting the quantification of LC3B protein levels. (**b**–**d″**) LC3B in placentas of control, L-NAME-treated, or L-NAME + esomeprazole-treated mice were evaluated by immunofluorescence staining. (**e**) The protein levels of phospho-AMPKα, AMPKα, PPARγ, SirT1, mTOR, and phospho-mTOR in placentas of control, L-NAME-treated, or L-NAME + esomeprazole-treated mice were detected by Western blot. (**f**–**f⁗**) Quantification of the LC3B, HIF1α, pAMPKα/AMPKα, PPARγ, and phospho-mTOR/mTOR in placentas of control, L-NAME-treated, or L-NAME + esomeprazole-treated mice, *N* = 3 mice per group, *P < 0.05, **P < 0.01

To understand the molecular mechanisms by which esomeprazole inhibits autophagy in L-NAME-treated placenta, we first analyzed the protein level of LC3B. Western blot analysis revealed that LC3B protein levels were significantly elevated in the PE group compared to the control group, whereas esomeprazole treatment potently reduced the LC3B levels (Fig. 8a, a′). Comparable results were obtained comparing the LC3B expression level in control, diseased, or treated placentas based on immunofluorescence staining-based assays (Fig. 8b–d″). More importantly, we found that PPARγ protein levels were also decreased in the PE mice placentas (*P* < 0.05). Interestingly, the phosphorylation of AMPKα was elevated in the PE group; however, the phosphorylation of mTOR was significantly inhibited (*P* < 0.05). Esomeprazole inhibited the phosphorylation of AMPKα, whereas it increased the phosphorylation of mTOR (Fig. 8e–f‴).

### Discussion

PE has negative consequences on maternal and fetal health during pregnancy, including increased perinatal mortality, preterm births, infants who are considered small for gestational age, high rates of cesarean deliveries, and other adverse outcomes, even in later postnatal periods (Antza et al. 2018; Gu et al. 2019). The relevant mechanism is not yet fully elucidated. Although many factors impact preeclampsia—including some not yet identified—recent evidence suggests that insufficient trophoblast cell invasion causes uterine spiral artery remodeling disorder and shallow placental implantation (Mol et al. 2016b). This leads to placental ischemia and hypoxia, followed by excessive autophagy.

We found that HIF-1α was elevated in PE placenta; this only occurs for a long time under hypoxic conditions. In addition to the resulting placental ischemia and hypoxia, bioactive factors released by the mother into the blood circulation cause a systemic inflammatory reaction and vascular endothelial injury, inducing clinical symptoms of PE. Soluble Fms-like tyrosine kinase-1 (sFlt-1), an antagonist of VEGF, has been shown to induce preeclampsia-like disease in rodents (Hastie et al. 2019). This also has been confirmed in our animal experiments. The placenta is undoubtedly involved in the pathogenesis, considering that termination of the pregnancy can eliminate the disease.

In vitro, our findings indicate that autophagy was increased in PE placenta. During the early pregnancy period, a low-oxygen environment in the placenta is beneficial for the differentiation of trophoblast cells from trophoblastic stem cells, promoting trophoblastic cell migration and remolding spiral arteries during this period in humans (Nakashima et al. 2017). Under these conditions, autophagy is activated (Yamanaka-Tatematsu et al. 2013). Proper autophagy is necessary to regulate protein quality control and maintain intracellular homeostasis (Levine and Kroemer 2019). However, excessive autophagy induced by long-term anoxia can lead to cell death and affect placental function. To better simulate the pathological conditions of preeclampsia, cells were treated by hypoxia and L-NAME treatment for 48 h, followed by the rapid activation of autophagy. Excessive autophagy disrupts invasion and vascular remodeling under hypoxia. We found that, with increased HIF-1α expression, placental autophagy was enhanced; the autophagy manifested as elevated LC3B levels. Excessive autophagy is accompanied by proliferation arrest and the transformation to the pro-inflammatory phenotype (Nakashima et al. 2017). The placental-autophagy disorder releases abundant toxic factors that damage maternal vasculature.

Autophagy is controlled by multiple signaling pathways (Lamark et al. 2017). We found—in combination with increased HIF-1α expression—an increased phosphorylation of AMPKα via SIRT1 activation, the reduced phosphorylation of mTOR, and diminished activity, resulting in the enhancement of placental autophagy. This manifested as elevated LC3B levels. AMPK is best known as a protein kinase that regulates energy metabolism (Kim and Lee 2014). It is expressed in various metabolism-related organs and can be activated via various stimuli, including cellular stress, exercise, and numerous hormones and substances that affect cellular metabolism. AMPK activation is dependent on the deacetylation of the lysine of liver kinase B1 residue by SIRT1 (Ganesan et al. 2017). In our study, SIRT1 also was found to be highly activated in the PE placenta. SIRT1 exerts its cellular autonomic function by regulating many transcription factors, such as p53, in the nucleus. SIRT1 controls cellular-repair mechanisms, such as autophagy and mitochondrial biogenesis. The phosphorylation of AMPKα governs the formation of autophagy vesicles by the PI3KC3 complex (Hurley and Young 2017). Additionally, we found that the SIRT1-dependent activation of AMPK downregulates mTOR, which then initiates a cellular stress response that includes autophagy (Xu et al. 2012). The phosphorylation of mTOR increases the formation of the mTORC1 and mTORC2 complexes. The inhibition of mTORC1 activates autophagy, whereas its activation reduces autophagy. Our study provides evidence that, in the placenta, AMPK and mTOR are engaged in regulating autophagy. We found that excessive autophagy decreased the invasion of HTR8/SVneo. In addition, the hypoxia-induced phosphorylation of AMPKα reduces the expression of PPARγ. PPARγ is regulated in part by HIF-1α (Tache et al. 2013). PPAR-γ is an important regulator of spiral artery development and placental function (Liu et al. 2017). Its participation has been noted in the pathogenesis of intrauterine growth retardation and preeclampsia. Evidence suggests that PE placental autophagy is regulated by hypoxia-induced AMPKα phosphorylation.

Esomeprazole, a proton-pump inhibitor, is a drug widely used to treat gastroesophageal reflux disease, a common condition associated with pregnancy (Galmiche et al. 2011). It has been shown that patients receiving esomeprazole experience less gestational hypertension and lower plasma ENG and sFLT-1 levels (Onda et al. 2017). Our animal experiments confirm this. A prospective Dutch cohort study treated 430 pregnant women with preeclampsia with PPIs, alpha methyldopa, steroid hormones, ferrous fumarate, polyethylene glycol, and nifedipine. This study found that PPIs reduced the level of sFlt-1 level and sENG and prolonged the week of pregnancy effectively (Saleh et al. 2017). Previous research also found that PPIs could upregulate the key placental protective enzyme, heme-oxygenase 1, and then improved the maternal antioxidant-defense function. Furthermore, esomeprazole also mitigates tumor necrosis factor-α–induced endothelial dysfunction. Esomeprazole reduces expression of endothelial vascular cell adhesion molecule 1, prevents leukocyte adherence to endothelium, and promotes angiogenesis (Brownfoot et al. 2016; Kaitu'u-Lino et al. 2017). In our PE mice model, we also found that esomeprazole decreased sFlt-1 serum levels. It has been reported that heme-oxygenase 1 (HO-1) may decrease sFlt-1 secretion (Cudmore et al. 2007), although Tong et al. came to the opposite conclusion (Tong et al. 2015). Moreover, researchers found combining esomeprazole with other drugs, such as metformin and sulfasalazine, in lower concentrations caused an additive reduction in sFlt-1 secretion in primary cytotrophoblasts, placental explants, and endothelial cells (Binder et al. 2020; Kaitu'u-Lino et al. 2018). Additionally, with treatment of esomeprazole, in the aortic tissues of pregnant L-NAME-treated mice, autophagy was inhibited (Zhang et al. 2021). This result is similar to ours, finding that placental autophagy was inhibited with the treatment of esomeprazole. Therefore, we hypothesize that esomeprazole treatment may reduce the autophagy of trophoblasts and endothelial cells, thereby preventing the release of sFLT-1 into maternal blood. In vitro*,* the autophagy of HTR-8/SVneo cells and HUVEC cells induced by hypoxia and L-NAME treatment was inhibited by the esomeprazole treatment. Under the esomeprazole treatment, the expression of HIF-1α and the activation of AMPK were reduced in vivo and in vitro. This constitutes evidence that cell hypoxia and metabolic abnormalities were relieved. Esomeprazole may prevent autophagy induced by hypoxia.

## Conclusion

The PE placenta experiences excessive autophagy as a result of long-term anoxic conditions. This autophagy contributes to PE symptoms. Autophagy is regulated by the SIRT1/AMPKα-mTOR pathway, upon which esomeprazole acts. Thus, esomeprazole can suppress preeclampsia-like symptoms by inhibiting excessive placental autophagy in PE, acting via the SIRT1/AMPKα-mTOR pathway. Furthermore, esomeprazole may increase the expression of PPARγ, thereby protecting placental function.
